# Effects of feeding CLOSTAT (*Bacillus subtilis* PB6) on the clinical health, performance, and carcass characteristics of feedlot steers^1^

**DOI:** 10.1093/tas/txad047

**Published:** 2023-05-11

**Authors:** Will R Ryan, Elizabeth S DeSocio, Maggie E Youngers, Caleb G Lockard, Chris J Richards, Sara J Trojan, Jerilyn E Hergenreder, Blake K Wilson

**Affiliations:** Department of Animal and Food Sciences, Oklahoma State University, Stillwater, OK 74078, USA; Department of Animal and Food Sciences, Oklahoma State University, Stillwater, OK 74078, USA; Department of Animal and Food Sciences, Oklahoma State University, Stillwater, OK 74078, USA; Department of Animal and Food Sciences, Oklahoma State University, Stillwater, OK 74078, USA; Department of Animal and Food Sciences, Oklahoma State University, Stillwater, OK 74078, USA; Peak Beef Nutrition and Management Consulting, LLC, Casper, WY 82604, USA; Kemin Industries, Inc., Des Moines, IA 50317, USA; Department of Animal and Food Sciences, Oklahoma State University, Stillwater, OK 74078, USA

**Keywords:** *Bacillus subtilis* PB6, bovine respiratory disease, calf health, direct-fed microbial, feed efficiency

## Abstract

The objectives of this experiment were to evaluate the effects of feeding *Bacillus subtilis* PB6 on clinical health, performance, and carcass characteristics of feedlot steers. *Bos indicus* crossbred steer calves (*n* = 397; 342 kg initial body weight [BW]) were randomly assigned to pens by initial BW; pens (*n* = 24) were randomly assigned to one of two of the following experimental treatments: 1) no supplemental dietary direct-fed microbial, control (CON; *n* = 12 pens) or 2) 13 g/steer daily *B. subtilis* PB6 (CLO; CLOSTAT, Kemin Industries, Des Moines, IA; *n* = 12 pens). Steers were housed in 12.2 × 30.5 m soil-surfaced pens; pen served as the experimental unit. The percentage of cattle treated once or twice for bovine respiratory disease (BRD) did not differ among treatments (*P* ≥ 0.27); BRD mortality also did not differ between CON and CLO (*P* = 0.34). During the receiving period, final BW (*P* = 0.97), average daily gain (ADG; *P* = 0.91), dry matter intake (DMI; *P* = 0.77), and gain:feed (*P* = 0.79) were not different among treatments. There was a tendency (*P* = 0.09) for CLO-supplemented steers to be 14% more efficient from days 0 to 14 of the receiving period. Final BW, overall finishing phase ADG, and DMI did not differ by treatment (*P ≥* 0.14); ADG was 0.14 kg greater for CLO than CON (*P* = 0.03) from days 29 to 56 of the finishing period. Gain: feed tended (*P* = 0.07) to be 7% greater (0.144 vs. 0.141) for CLO than CON throughout the duration of the finishing period, and 6.7% greater (*P* = 0.08; 0.152 for CLO vs. 0.150 for CON) for the entirety of the experiment. Carcass traits did not differ among treatments (*P ≥* 0.31). The results of this experiment suggest that supplementing 13 g/steer daily *B*. *subtilis* PB6 may improve feed efficiency in feedlot cattle.

## INTRODUCTION

In recent years, the cattle feeding industry has attempted to reduce the use of antimicrobials due to consumer demand and antimicrobial resistance concerns (Krehbiel et al., 2003; Wilson and Krehbiel, 2012). Accordingly, research seeking to understand the application and economic feasibility of nutraceuticals in the feedlot industry has expanded. Newly received cattle in a feedlot are exposed to a multitude of stressors including weaning, transportation, environmental changes, and commingling. These stressors can compromise the immune system of calves, leading to bovine respiratory disease (BRD; Krehbiel et al., 2003; Wilson et al., 2017). Morbidity and mortality resulting from BRD continue to be the most economically significant problems facing the feedlot industry (Duff and Galyean, 2007; Wilson et al., 2017; Peel, 2020). Incorporating nutraceuticals into diets of feedlot cattle, especially during the receiving period, may be advantageous, particularly as a management tool to aid in improving animal performance and decreasing morbidity (Duff and Galyean, 2007; Ballou et al., 2019;). Research also suggests the supplementation of nutraceuticals in diets of newly received cattle may have the potential to decrease the dietary adaption period, allowing cattle to consume a more energy-dense diet earlier in the finishing period (Swanson et al., 2018). Finally, some data suggest that feeding nutraceuticals may increase performance of finishing cattle (NASEM, 2016).

*Bacillus subtilis* PB6 (CLOSTAT, Kemin Industries, Des Moines, IA) is a naturally occurring, spore-forming organism. Previous research has reported that *B. subtilis* PB6 can improve intestinal health by inhibiting the growth of pathogenic bacterial strains, maintaining the balance of commensal bacterial microflora, and reducing inflammation (Kim et al., 1998; Teo and Tan, 2005, 2006; Selvam et al., 2009).

The efficacy of *B. subtilis* PB6 in cattle has been demonstrated through previous research and has been shown to reduce the severity of salmonellosis (Broadway et al., 2020), decrease BRD morbidity (Smock et al., 2019; Word et al., 2022), and improve profitability in the feedyard (Word et al., 2022). This experiment was conducted in 2017 and was the first applied study conducted with *B. subtilis* PB6 in feedlot cattle; therefore, the objectives of this experiment were to evaluate the effects of supplementing 13 g/steer daily of *B. subtilis* PB6 on the clinical health, performance, and carcass characteristics of feedlot steers. We hypothesized that feeding *B. subtilis* PB6 would improve the aforementioned variables in feedlot steers.

## MATERIALS AND METHODS

All procedures for the present experiment were approved by the Oklahoma State University Institutional Animal Care and Use Committee (ACUP # AG-17-13).

### Animal Processing, Housing, and Body Weight (BW) Collection

In mid-September 2017, *Bos indicus* crossbred steer calves (*n* = 397) arrived in three separate shipments within 4 d to the Willard Sparks Beef Research Center in Stillwater, OK, from ranch units in Florida. Steers were individually weighed and received a unique individual ear tag immediately after arrival. Steers were allowed to rest for 24 to 48 h after arrival, prior to being processed. On day 0 of the experiment, steers were administered a clostridial bacterium/toxoid vaccine (Vision 7 with SPUR; Merck Animal Health, Rahway, NJ), and treated for external (StandGuard; Elanco Animal Health, Greenfield, IN) and internal (Safe-Guard; Merck Animal Health) parasites. Steers also received an individual color-coded experimental treatment tag and implant (REVALOR-IS; Merck Animal Health). For the entirety of the experiment, steers were housed in 12.2 × 30.5 m soil-surfaced pens, with a 12.2-m solid concrete bunk and a 12.2 × 3.7-m concrete apron. Water tanks (model J 360-F; Johnson Concrete, Hastings, NE) were shared by pens on the same experimental treatment to prevent any cross-contamination of experimental treatments via shared water source. Concrete bunk dividers were also used between pens to prevent cross-feeding and experimental treatment contamination. Twenty-four pens were utilized for this experiment resulting in 12 pens per treatment with 15 to 20 steers per pen. The number of steers per pen was based on shipment size and arrival date. The receiving period length was adjusted so all steers started the finishing period and began diet transition on the same date regardless of arrival date, corresponding to 61, 60, and 57 d receiving periods for shipment groups 1, 2, and 3, respectively. Steers were weighed every 14 d during the receiving period and every 28 d throughout the finishing period. Steers were re-implanted (REVALOR 200; Merck Animal Health) on January 16, 2018 (day 28 of the finishing phase). Steers were fed ractopamine-hydrochloride (Optaflexx; Elanco Animal Health) at the rate of 300 mg/steer daily from days 141 to 169 of the finishing period and underwent a 48-h ractopamine-hydrochloride withdrawal prior to harvest.

### Experimental Treatments

Pens were pairwise assigned to treatments in a randomized complete block design. Treatments included: 1) no supplemental dietary direct-fed microbial, control (CON) or 2) 13 g/steer daily *B. subtilis* PB6 (CLO; CLOSTAT, Kemin Industries). The CON treatment consisted of a top-dressed supplement, containing only ground corn and wheat middlings fed at a rate of 0.227 kg/steer daily. The CLO treatment included 13 g/steer daily, CLOSTAT to provide 9,500,000 CFU/g in the same ground corn and wheat middlings carrier supplement, top-dressed, and fed at the same rate as CON (0.227 kg/steer daily). The inclusion of CLOSTAT in the CLO experimental top-dress was at the expense of equal amounts of ground corn and wheat middlings (Table 1). Treatment supplements were individually bagged and color coded.

**Table 1.** Ingredient composition of top-dress supplements containing the experimental treatments^1^

	Treatment	
Item, dry matter %	CON^2^	CLO^3^
Corn, ground	50.00	47.14
Wheat middlings	50.00	47.14
CLOSTAT	—	5.73

^1^Treatments were top-dressed daily at 0.227 kg/steer daily.

^2^Control supplement.

^3^ *B. subtilis* PB6 (CLOSTAT, 9,500,000 CFU/g; Kemin Industries, Inc.) to provide 13 g/steer daily of CLOSTAT.

### Feed and Bunk Management

During the receiving period, steers were fed a prairie hay, alfalfa hay, and Sweet Bran (Cargill Inc., Dalhart, TX) based receiving diet (Table 2). After the receiving period, steers were transitioned over 21 d to a high-concentrate finishing diet for the remainder of the experiment. The finishing diet contained 8% roughage and was primarily composed of dry-rolled corn and Sweet Bran (Cargill Inc.; Table 2). Diets were formulated to meet or exceed all nutrient requirements for receiving and finishing steers (NASEM, 2016).

**Table 2.** Composition of common receiving and finishing diets^1^

Item	Receiving	Finishing
Ingredient, % dry matter		
Prairie hay	25.00	8.00
Alfalfa hay	20.00	—
Sweet Bran^2^	45.00	20.00
Dry-rolled corn	—	62.00
Dry supplement^3^	5.00	5.00
Liquid supplement^4^	5.00	5.00
Analyzed nutrient composition (DM basis)^5^		
Dry matter, % (as-fed basis)	72.75	76.38
NE_m_, Mcal/kg	1.47	2.18
NE_g_, Mcal/kg	0.88	1.51
Total digestible nutrients, %	64.95	88.03
Crude protein, %	17.95	13.32
Acid detergent fiber, %	27.70	9.95
Calcium, %	0.79	0.52
Phosphorus, %	0.65	0.49
Magnesium, %	0.33	0.21
Potassium, %	1.42	0.76

^1^All values except dry matter are presented on a dry matter basis.

^2^Sweet Bran (Cargill).

^3^Dry supplement was formulated to contain (% DM basis) 42.63% ground corn, 27.14% calcium carbonate, 20.60% wheat middlings, 6.51% urea, 0.92% salt, 0.49% magnesium oxide, 0.47% zinc sulfate, 0.15% manganous oxide, 0.12% copper sulfate, 0.08 selenium, 0.29% vitamin A (30,000 IU/g), 0.09 vitamin E (500 IU/g), 0.008% vitamin D (30,000 IU/g), 0.302% monensin (Rumensin 90; Elanco Animal Health), and 0.186% tylosin (Tylan 40; Elanco Animal Health).

^4^Liquid supplement was formulated to contain (% DM basis) 45.85% corn steep, 36.17% corn molasses, 6.00% hydrolyzed vegetable oil, 5.46% 80 VOP/20 oil, 5.19% water, 1.23% urea, 55% solution, and 0.10% xanthan gum (Westway Feed Products; Tomball, TX).

^5^Feed samples were analyzed for nutrient composition and energy values were calculated by an independent laboratory (ServiTech Laboratories).

Every morning at 0530 h feed bunks were visually evaluated by trained facility personnel. The evaluation of the bunk was used to adjust the feed amount offered daily in an attempt to allow 0.045 kg of feed or less to remain in the feed bunk (modified slick bunk approach). Feed bunks were also evaluated every evening at 1730 h. Steers were fed twice daily at 0745 and 1100 h. The daily feed offering was not adjusted prior to 1100 h (feed calls were made once per day). Steers were fed respective experimental top-dress supplements once daily at 0745 h. No experimental supplements were fed during the second feeding. Experimental treatment top-dress supplements (CON and CLO) were individually weighed into 24 individually numbered, color coded, containers daily. Immediately following the morning feed delivery, the appropriate experimental top-dress was delivered to the appropriate pen, dispersed evenly onto the feed, and hand-mixed into the complete ration in the bunk with a color-coded designated pitchfork to prevent dietary cross-contamination during mixing. Steers were not allowed access to the feed bunk until the top-dress was fully mixed with complete ration. All pens were individually labeled and color coded to match the experimental treatments. Diets were mixed and fed from a pull-behind feed wagon (Roto-Mix 274-12B Forage Express; Dodge City, KS). Feed refusals were weighed back if feed remained in the bunk for two consecutive days, and on mornings of weigh days or following inclement weather. Diet and ingredient samples were taken twice per week, and the dry matter content of the diets and dietary ingredients was determined by samples drying in a forced-air oven at 60 °C for 48 h. Dried diet samples were composited monthly and sent to a commercial lab for nutrient analysis (Table 2; ServiTech Laboratories, Dodge City, KS).

### Harvest and Carcass Characteristics

On day 231 of the experiment, steers were transported 435 km on 12 trucks to a commercial abattoir in Dodge City, KS, for harvest. All carcass data were collected by trained individuals from West Texas A&M Beef Carcass Research Center. Data collected for each steer included hot carcass weight (HCW), dressing percentage, 12th rib-fat thickness, longissimus muscle area, USDA yield grade, marbling score, USDA quality grade, and liver score.

### Animal Health

Each morning throughout the experiment, trained personnel visually monitored steers for clinical signs of BRD and lameness using a modified DART system described by Step et al. (2008) and Wilson et al. (2015). The visual signs used to “pull” a steer for suspected BRD included depression, abnormal appetite, and respiratory signs. Severity scores ranged from 0 to 4 and were assigned to steers in accordance with the presence and severity of observed clinical signs as follows: (0) no clinical signs, (1) mild clinical signs, (2) moderate clinical signs, (3) severe clinical signs, or (4) a moribund animal. Steers that received a severity score of 1 to 4 were removed from the pen for further evaluation. After obtaining a rectal temperature measurement from the steer using a thermometer (GLA M-500; GLA Electronics, San Luis Obispo, CA), determination for treatment administration was the following: 1) steers that received a severity score of 1 or 2 were administered an antimicrobial with a rectal temperature ≥40.0 °C and 2) steers that received a severity score of 3 or 4 were administered an antimicrobial regardless of rectal temperature.

BW was obtained prior to antimicrobial administration to determine antimicrobial dosage. The schedule of antibiotic administration included: 1) initial treatment, tildipirosin (Zuprevo; Merck Animal Health), administered subcutaneously on the left side of the neck; 2) secondary treatment could not be administered within 120-h postinitial antimicrobial administration; for steers that warranted a secondary treatment, florfenicol (Nuflor; Merck Animal Health) was administered subcutaneously on the right side of the neck; and 3) tertiary treatment could not be administered within 120-h postsecondary antimicrobial administration, for steers that warranted tertiary treatment, ceftiofur crystalline-free acid (EXCEDE; Zoetis, Parsipanny, NJ) was administered subcutaneously at the base of the left ear. Steers could receive a maximum of three antimicrobials. After antimicrobial administration, steers were returned to the home pen.

### Statistical Analysis

Deads and removed animals were excluded from the statistical analysis for all performance and carcass data (deads and removals out data). Any steers that either died or were removed during the receiving period had the total number of head days of dry matter intake (DMI) subtracted from the pen. As all steers removed during the receiving period were losing BW at the time of removal, DMI was subtracted from the pen based on individual calculated maintenance energy intake using dietary NE_m_ concentration and the following equation: NE_m_ = 0.077 SBW^0.75^ (NASEM, 2016). Any steers that died or were removed during the finishing period, had the total head days of DMI removed as previously described; however, the DMI subtracted for steers removed during finishing was determined to be the average of the pen for that period, as all steers were gaining BW at the time of death or removal.

A randomized complete block design was used for this experiment. Pen served as the experimental unit, and block (*n* = 12) was included as a random effect with BW serving as the blocking factor. Animal performance and carcass data, excluding quality grade and liver abscess data, were analyzed using the PROC MIXED procedure of SAS 9.4 (SAS Inst. Inc., Cary, NC). Clinical health, quality grade, and liver abscess data were analyzed using PROC GLIMMIX procedure (SAS Inst. Inc.). Significance was declared if *P* ≤ 0.05, and tendencies were declared if 0.05 < *P* ≤ 0.10.

## RESULTS AND DISCUSSION

### Clinical Health

The effects of feeding *B*. *subtilis* PB6 on receiving calf health are reported in Table 3. There was no difference among experimental treatments for first (*P* = 0.27) or second BRD antimicrobial administration percentages (*P* = 0.55). Similarly, there was no difference in average rectal temperatures (*P* = 0.50) or severity scores (*P* = 0.24) between CON and CLO. There was no difference (*P* = 0.34) between experimental treatments for the percentage of mortalities and removals due to BRD. However, there was a difference in percentage of mortalities and removals due to lameness and complications from toe abscesses among the two treatments. The CON treatment had a lower percentage (*P* = 0.02) of mortalities and removals related to complications from toe abscesses per pen compared to CLO (2.01% vs. 5.77% for CON and CLO, respectively). Care should be taken in interpreting the mortality and removal results as only one steer died during the experiment due to confirmed BRD. The remainder of the animals that died or were removed from the experiment were a result of lameness, complications from toe abscesses, and other non BRD-related health complications. While there was a difference between the experimental treatments for non BRD-related mortalities and removals, this is not believed to be a result of the experimental treatments, as to our knowledge there is nothing reported in the literature indicating the supplementation of CLO could result in toe abscesses or lameness issues. Similar experiments by Smock et al. (2019) and Word et al. (2022) make no mention of incidence of toe abscesses or lameness.

**Table 3.** Effects of feeding *B. subtilis* PB6 on clinical health of feedlot steers

Item	Treatment^1^		SEM	*P*-value
	CON	CLO		
1st BRD antimicrobial treatment^2^, %	12.50	8.39	2.53	0.27
2nd BRD antimicrobial treatment^3^, %	1.97	1.04	1.08	0.55
Temperature at BRD treatment, °C	40.4	40.5	0.14	0.50
Severity score at BRD treatment^4^	1.94	2.27	0.22	0.24
BRD-related mortalities and removals^5^, %	0.00	0.52	0.37	0.34
Other mortalities and removals^6^, %	2.01	5.77	1.32	0.02

^1^Fed a supplement at 0.227 kg/steer daily containing only corn and wheat middlings (CON) or the CON with added (CLOSTAT, 9,500,000 CFU/g of *B. subtilus*; Kemin Industries, Inc.) to provide 13 g/steer daily of CLOSTAT (CLO).

^2^Percentage of steers per pen that received antimicrobial treatment for bovine respiratory disease (BRD).

^3^Percentage of steers per pen that received two or more antimicrobial treatments for BRD.

^4^Severity scores were assigned based upon a modified DART system described by Wilson et al. (2016) and Step et al. (2008). Numerical severity scores ranged from 0 to 4 and were assigned to steers in accordance with the presence and severity of observed clinical signs as follows: (0) no clinical signs/clinically normal animal, (1) mild clinical signs, (2) moderate clinical signs, (3) severe clinical signs, or (4) a moribund animal.

^5^Percentage of BRD mortalities and removals. Only one mortality was due to confirmed BRD.

^6^Percentage of mortalities and removals as a result of lameness, complications with toe abscesses, and other issues.

Possible reasons for the elevated number of cattle removed due to lameness and complications from toe abscesses may be due to several factors. As mentioned previously, the cattle used in this experiment experienced a significant weather event immediately prior to arrival. In addition, the cattle were of *B. indicus* influence and had marginal exposure to human handlers prior to arrival at the feedlot. As a result, the steers were extremely excitable and had large flight zones, which attributed to multiple slips and falls in the pens and in the processing area. We believe that the majority of the lameness and hoof issues that occurred were ultimately due to cattle temperament, the frequent handling of flighty cattle on concrete surfaces, and potentially soft hooves caused by a wet environment immediately prior to the experiment, and not a result of experimental design or experimental treatment.

CLOSTAT contains a patented strain of *B. subtilis* PB6, an active microbial present in the intestinal tract of healthy chickens (Teo and Tan, 2005). The effects of *B. subtilis* on pathogen inhibition and intestinal health have been documented through multiple modes of action. Direct and indirect inhibition of several gram-positive and gram-­negative pathogenic bacterial strains occurs through the action of secondary metabolites of PB6, while commensal bacterial populations are maintained (Teo and Tan, 2005, 2006). It has also been demonstrated that secondary metabolites of PB6 inhibit certain inflammatory pathways (Kim et al., 1998; Selvam et al., 2009; Goetz et al., 2022).

Previous literature has demonstrated positive health outcomes and improved feedyard profitability by feeding *B. subtilis* PB6. Supplementing *B*. *subtilis* PB6 reduced the severity of *Salmonella* challenge in calves through an improved immune response associated with increased serum concentrations of white blood cells, lymphocytes, and acute phase proteins, and a lower overall febrile response than the nonsupplemented control calves (Broadway et al., 2020). *B. subtilis* PB6 supplemented calves had lower *Salmonella* concentrations 48-h postchallenge in the jejunum, ileum, and transverse colon tissues than control (Broadway et al., 2020). Furthermore, Smock et al. (2019) reported a reduction in initial and secondary BRD treatment rates in high-risk feeder steers fed *B. subtilis* PB6 and a decrease in fecal *Salmonella* prevalence over control steers. In a commercial feedyard, supplementing *B. subtilis* PB6 to yearling steers reduced the overall treatment rate by 29%, lowered the initial BRD treatment rate by 39.6%, resulted in fewer cattle receiving a second BRD treatment, and fewer cattle removed from the experiment (Word et al., 2022). Further, when considering the losses of dead and railer cattle in the aforementioned experiment, authors projected a 5.55:1 return on investment by feeding *B. subtilis* PB6.

Although there were no differences in first or second antimicrobials administered for BRD in the present experiment, steers supplemented with CLO had numerically lower values for first (4.11% less) and second (0.93% less) antimicrobials administered for BRD. It should be noted that overall morbidity (10.4%) was less than expected based on the risk classification of the steers used in the current experiment (Wilson et al., 2017). The relatively lower rates of overall morbidity observed may partially explain the lack of statistical differences observed between experimental treatments for animal health variables. Presumably, there was not enough statistical power in this experiment to detect these differences when overall morbidity was much less than expected.

### Receiving Performance

Effects of feeding CLO on receiving feedlot steer performance are reported in Table 4. Throughout the receiving period (61 d for group 1, 60 d for group 2, and 57 d for group 3), there were no differences in final BW (*P* = 0.97), overall receiving average daily gain (ADG; *P* = 0.91), overall receiving DMI (*P* = 0.77), or overall receiving gain: feed (*P* = 0.79). There was a tendency (*P* = 0.09) for CLO steers to be more efficient than the CON steers from days 0 to 14 of the receiving period (0.206 vs. 0.177, respectfully). This tendency may be partially explained by the numerical difference in morbidity between the experimental treatments, as 41.0% of the total morbidity (4.28% out of the 10.4%) occurred during the first 14 d. Smock et al. (2019) reported that high-risk feeder calves supplemented with *B. subtilis* PB6 had increased ADG and DMI during a 56-d receiving period. Historical research by Gill et al. (1987) reported that the ADG and G:F were increased during the receiving period for calves being supplemented an *Enterococcus faecium* and *Lactobacillus acidophilus* combination direct-fed microbial product. Sun et al. (2010) concluded that dairy calves being supplemented an alternative strain of *B*. *subtilis* had improved ADG and gain:feed; the authors further stated that the weaning age of the supplemented calves was accelerated without adverse effects. Responses to nutraceutical supplementation are variable in the literature as compounds differ by type, mode of action, and application (Ballou et al., 2019). In a review of direct-fed microbial compounds by Krehbiel et al. (2003), authors indicated that in some experiments feeding a direct-fed microbial to newly received calves improved feed efficiency by 2.0% and increased ADG by 2.5% to 5.0%. However, these improvements are not consistent across other experiments with direct-fed microbials (Wilson and Krehbiel, 2012). Wilson et al. (2016) reported no difference in ADG or gain:feed due to supplementation of a direct-fed microbial but mentioned that differences may have not been able to be detected due to experimental design (six heifers per pen). Wilson et al. (2016) further explained that improvements in ADG and gain:feed reported for direct-fed microbial supplemented calves in the literature are generally small (<5%), and these differences would be difficult to detect in a small pen setting.

**Table 4.** Effects of feeding *B. subtilis* PB6 on receiving performance of feedlot steers

Item^1^	Treatment^2^		SEM^3^	*P*-value
	CON	CLO		
Body weight^4^, kg				
Day 0	253	253	6.0	0.78
Day 14	264	266	6.1	0.45
Day 28	286	285	6.6	0.77
Day 42	311	312	6.6	0.76
Final^5^	342	342	6.4	0.97
Average daily gain, kg				
Days 0 to 14	0.82	0.94	0.06	0.17
Days 15 to 28	1.53	1.40	0.10	0.14
Days 29 to 42	1.82	1.91	0.07	0.32
Days 43 to final	1.71	1.66	0.11	0.55
Days 0 to final	1.49	1.50	0.03	0.91
Dry matter intake, kg				
Days 0 to 14	4.6	4.5	0.08	0.39
Days 15 to 28	7.4	7.8	0.23	0.52
Days 29 to 42	9.7	9.6	0.22	0.45
Days 43 to final	10.4	10.4	0.19	0.63
Days 0 to final	8.2	8.2	0.14	0.77
Gain:feed				
Days 0 to 14	0.177	0.206	0.012	0.09
Days 15 to 28	0.212	0.184	0.016	0.18
Days 29 to 42	0.189	0.200	0.007	0.15
Days 43 to final	0.163	0.160	0.009	0.65
Days 0 to final	0.182	0.183	0.003	0.79

^1^Data are presented on a deads and removals out basis. Head days were removed from the pen and feed intake was removed using a calculated maintenance dry matter intake (NASEM, 2016).

^2^Treatments included: steers fed a supplement at 0.227 kg/steer daily containing only corn and wheat middlings (CON) or the CON with (CLOSTAT 9,500,000 CFU/g of *B. subtilus*; Kemin Industries, Inc.) to provide 13 g/steer daily of CLOSTAT (CLO).

^3^Standard error of the mean.

^4^Body weight analyzed with a 4% pencil shrink.

^5^Final receiving body weight was recorded on day 61 for group 1 (shipment group 1), day 60 for group 2 (shipment group 2), and day 57 for group 3 (shipment group 3).

### Finishing Performance

Effects of feeding CLOSTAT on finishing feedlot steer performance are reported in Table 5. There was no difference in final BW (*P* = 0.40) at the end of the finishing period. There was an improvement in ADG from days 29 to 56 of the finishing phase for CLO compared with CON (*P* = 0.03), but no differences in overall ADG (*P* = 0.33) or overall DMI (*P* = 0.48). Compared with CON, cattle supplemented with CLO tended to have increased efficiency from days 29 to 56 of the finishing period (*P* = 0.06), a 7% improvement in gain:feed for the entire finishing period (*P* = 0.07), and a 6.7% improvement over the course of the entire experiment (combined receiving and finishing periods; *P* = 0.08). Smock et al. (2019) did not observe an effect of *B. subtilis* PB6 supplementation on finishing performance. Similarly, feedlot performance was not affected by *B. subtilis* PB6 supplementation to yearling steers (Smith et al., 2021). Yet, supplementing *B. subtilis* PB6 to feedlot steers in a commercial feedyard tended to improve live cattle performance on deads and removals included basis (Word et al., 2022).

**Table 5.** Effects of feeding *B. subtilis* PB6 on finishing performance of feedlot steers

Item^1^	Treatment^2^		SEM^3^	*P*-value
	CON	CLO		
Body weight^4^, kg				
Day 0^5^	342	342	6.4	0.97
Day 28	387	387	6.8	0.97
Day 56	423	427	6.7	0.14
Day 84	473	475	7.2	0.31
Day 112	511	514	6.9	0.25
Day 140	551	554	6.9	0.32
Final^6^	600	603	7.0	0.40
Average daily gain, kg				
Days 0 to 28	1.60	1.61	0.04	0.87
Days 29 to 56	1.30	1.44	0.04	0.03
Days 57 to 84	1.76	1.72	0.05	0.50
Days 85 to 112	1.37	1.36	0.04	0.90
Days 13 to 140	1.44	1.45	0.03	0.88
Days 141 to final^7^	1.66	1.68	0.05	0.74
Days 0 to final	1.52	1.54	0.01	0.25
Overall^8^	1.51	1.52	0.01	0.33
Dry matter intake, kg				
Days 0 to 28	10.4	10.3	0.18	0.70
Days 29 to 56	10.0	10.2	0.18	0.35
Days 57 to 84	10.5	10.4	0.14	0.62
Days 85 to 112	10.9	10.7	0.15	0.22
Days 113 to 140	11.2	11.1	0.14	0.33
Days 141 to final	11.9	11.7	0.13	0.15
Days 0 to final	10.8	10.7	0.13	0.46
Overall	10.1	10.0	0.12	0.48
Gain:feed				
Days 0 to 28	0.155	0.156	0.004	0.82
Days 29 to 56	0.130	0.142	0.005	0.06
Days 57 to 84	0.168	0.164	0.004	0.52
Days 85 to 112	0.126	0.128	0.004	0.77
Days 113 to 140	0.129	0.131	0.003	0.60
Days 141 to final	0.139	0.144	0.004	0.35
Days 0 to final	0.141	0.144	0.001	0.07
Overall	0.150	0.152	0.001	0.08

^1^Data are presented on a deads and removals out basis. Head days were removed from the pen and feed intake was removed using a calculated maintenance dry matter intake (NASEM, 2016).

^2^Treatments included: steers fed a supplement at 0.227 kg/steer daily containing only corn and wheat middlings (CON) or the CON with (CLOSTAT 9,500,000 CFU/g of *B. subtilis*; Kemin Industries, Inc.) to provide 13 g/steer daily of CLOSTAT (CLO).

^3^Standard error of the mean.

^4^Body weight analyzed with a 4% pencil shrink.

^5^Day 0 of finishing was day 61 for group 1 (shipment group 1), day 60 for group 2 (shipment group 2), and day 57 for group 3 (shipment group 3).

^6^Final = Total days on experiment; 231 d for group 1 (shipment group 1), 230 d for group 2 (shipment group 2), and 227 d for group 3 (shipment group 3).

^7^A beta-agonist (Optaflexx; Elanco Animal Health) was fed during this period at the rate of 300 mg/steer daily. There was a 48-h beta agonist withdraw before slaughter.

^8^Overall = entire experiment including both the receiving and finishing periods.

### Carcass Characteristics

Effects of feeding CLOSTAT on carcass characteristics are reported in Table 6. Carcass characteristics, including HCW, dressing percentage, 12th-rib fat, longissimus muscle area, marbling score, USDA quality grade, and yield grade did not differ by treatment (*P* ≥ 0.36). These results are consistent with other studies with *B. subtilis* PB6 supplementation to feedlot cattle (Smock et al., 2019; Smith et al., 2021; Word et al., 2022). Likewise, Krehbiel et al. (2003) frequently reported the literature supports that direct-fed microbial supplementation is not likely to affect carcass traits. Others have also reported findings similar to those in the present experiment; Swanson et al. (2018) evaluated the effects of the finishing system, dietary adaption length, and direct-fed microbial supplementation on the growth performance and feeding behavior of feedlot steers but did not report an effect of the direct-fed microbial on carcass characteristics. However, Vasconcelos et al. (2008) combined data from 2 experiments using a total of 240 feedlot steers when supplementing a direct-fed microbial and reported a tendency for a quadratic response in marbling score and percentage of steers grading USDA Choice. Likewise, Simas de Oliveira Moreira et al. (2016) detected an increased marbling score when Nellore bulls were supplemented with a combination of calcium butyrate and *B*. *subtilis.* While carcass improvements may be possible, Krehbiel et al. (2003) concluded that these improvements were typically observed as a yield response (i.e., increased HCW).

**Table 6.** Effects of feeding *B. subtilis* on carcass characteristics of feedlot steers

Item	Treatment^1^		SEM^2^	*P-*value
	CON	CLO		
Hot carcass weight, kg	386	388	4.59	0.58
Dressing percentage, %^3^	64.4	64.3	0.19	0.47
12th-rib fat, cm	1.13	1.10	0.03	0.36
Longissimus muscle area, cm^2^	94.4	94.5	0.96	0.91
Marbling^4^	459	456	7.96	0.82
USDA yield grade	2.66	2.63	0.06	0.72
USDA quality grade				
Prime, %	3.0	0.9	1.35	0.31
Choice +, %	3.8	4.9	1.45	0.60
Choice, %	22.6	23.3	3.89	0.90
Choice −, %	46.1	45.6	3.61	0.92
Select, %	24.5	25.3	4.14	0.88
Liver abscess^5^				
O, %	83.6	81.3	3.60	0.59
A−, %	3.75	4.75	1.71	0.69
A+, %	7.93	10.14	2.33	0.40
Condemned, %	4.69	3.84	1.74	0.73

^1^Treatments included: steers fed a supplement at 0.227 kg/steer daily containing only corn and wheat middlings (CON) or the CON with (CLOSTAT 9,500,000 CFU/g of *B. subtilis*; Kemin Industries, Inc.) to provide 13 g/steer daily of CLOSTAT (CLO).

^2^Standard error of the mean.

^3^Calculated by dividing the hot carcass weight by final shrunk body weight.

^4^Marbling scores: 400 = small, 500 = modest.

^5^Perecentage of liver abscesses within pen: O = edible; A− = 1 to 2 small abscesses; A+ = 1 or more large active abscesses or adhesions or several smaller abscesses; condemned = contaminated (Elanco Animal Health).

## CONCLUSION

Similar experiments have reported improved clinical health and animal performance with *B*. *subtilis* PB6 supplementation to feedlot cattle. Further, *B*. *subtilis* PB6 supplementation has resulted in improvements in immunity and intestinal health in dairy calves. While a statistical improvement in clinical health was not observed in the current experiment, the supplementation of *B*. *subtilis* PB6 did improve the feed efficiency of feedlot steers.

## Notes

^1^ We thank the student employees of the Willard Sparks Beef Research Center for assisting with this experiment.

## References

[CIT0001] Ballou, M. A., E. M. Davis, and B. A. Kasl. 2019. Nutraceuticals an alternative strategy for the use of antimicrobials. Vet. Clin. Food Anim. 35:507–534. doi:10.1016/j.cvfa.2019.08.004. PMC7127241 31590900

[CIT0002] Broadway, R. P., J. A. Carroll, N. C. Burdick Sanchez, T. R. Callaway, S. D. Lawhon, E. V. Gart, L. K. Bryan, D. J. Nisbet, H. D. Hughes, J. F. Legako, et al. 2020. *Bacillus subtilis* PB6 supplementation in weaned Holstein steers during an experimental *Salmonella* challenge. Foodborne Pathog. Dis. 17:521–528. doi:10.1089/fpd.2019.2757. 32349549

[CIT0003] Duff, G. C., and M. L. Galyean. 2007. BOARD-INVITED REVIEW: recent advances in management of highly stressed, newly received feedlot cattle. J. Anim. Sci. 85:823–840. doi:10.2527/jas.2006-501. 17085724 PMC7109667

[CIT0004] Gill, D. R., R. A. Smith, and R. L. Ball. 1987. The effect of probiotic feeding on health and performance of newly-arrived stocker calves. Okla. Agr. Exp. Stn. MP-119:202–204. https://extension.okstate.edu/programs/beef-extension/research-reports/site-files/documents/1987/87-48.pdf

[CIT0005] Goetz, B. M., E. J. Mayorga, M. A. Aveyta, S. Rodriguez-Jimenez, J. Opgenorth, A. Freestone, G. M. Jakes, C. E. Moore, D. J. Dickson, J. E. Hergenreder, et al. 2022. Effects of *Bacillus subtilis* PB6 on metabolism and production parameters in transition dairy cows. J. Dairy Sci. 105:154. (Abstr.). 37641308 10.3168/jds.2023-23562

[CIT0006] Kim, K., S. Y. Jung, D. K. Lee, J. K. Jung, J. K. Park, D. K. Kim, and C H Lee. 1998. Suppression of inflammatory responses by surfactin, a selective inhibitor of platelet cytosolic phospholipase A2. Biochem. Pharmacol. 55:975–985. doi:10.1016/s0006-2952(97)00613-8. 9605421

[CIT0007] Krehbiel, C. R., S. R. Rust, G. Zhang, and S. E. Gilliland. 2003. Bacterial direct-fed microbials in ruminant diets: performance response and mode of action. J. Anim. Sci. 81:E120–E132. doi:10.2527/2003.8114_suppl_2E120x.

[CIT0008] NASEM. 2016. Nutrient requirements of beef cattle: eighth revised edition. Washington (DC): The National Academics Press. doi:10.17226/19014. 38386771

[CIT0009] Peel, D. S. 2020. The effect of market forces on bovine respiratory disease. Vet. Clin. Food Anim. 36:497–508. doi:10.1016/j.cvfa.2020.03.008. 32451038

[CIT0010] Selvam, R., P. Maheswari, P. Kavitha, M. Ravichandran, B. Sas, and C. N. Ramchand. 2009. Effect of *Bacillus subtilis* PB6, a natural probiotic on colon mucosal inflammation and plasma cytokines levels in inflammatory bowel disease. Indian J. Biochem Biophys. 46:79–85. https://pubmed.ncbi.nlm.nih.gov/19374258/. 19374258

[CIT0011] Simas de Oliveira, T., K. Oliveira Marques, K. C. Guimarᾶes, W. A. Marchesin, U. O. Bilego, and N. F. Freitas. 2016. Duodenal histology and carcass quality of feedlot cattle supplemented with calcium butyrate and *Bacillus subtilis*. Acta Sci., Anim. Sci. 38:61–67. doi:10.4025/actascianimsci.v38i1.27432.

[CIT0012] Smith, Z. K., P. R. Broadway, K. R. Underwood, W. C. Rusche, J. A. Walker, N. C. Burdick, J. A. Carroll, D. Lafleur, and J. E. Hergenreder. 2021. Evaluation of *Bacillus subtilis* PB6 on feedlot phase growth performance, efficiency of dietary net energy utilization, and fecal and subiliac lymph node *Salmonella* prevalence in spring placement yearling beef steers fed in southeastern South Dakota. Transl. Anim. Sci. 5:1–10. doi:10.1093/tas/txab002. PMC7881255 33604519

[CIT0013] Smock, T. M., K. Samuelson, H. A. Seiver, J. Hergenreder, and W. Rounds. 2019. PSIV- 15 effects of *Bacillus subtilis* PB6 and/or chromium propionate supplementation on health, performance, and blood parameters of high-risk cattle during the feedlot receiving period. J. Anim. Sci. 97:231–231. doi:10.1093/jas/skz258.470.30312406

[CIT0014] Step, D. L., C. R. Krehbiel, H. A. Depra, J. J. Cranston, R. W. Fulton, J. G. Kirkpatrick, D. R. Gill, M. E. Payton, M. A. Montelongo, and A. W. Confer. 2008. Effects of commingling beef calves from different sources and weaning protocols during a forty-two-day receiving period on performance and bovine respiratory disease. J. Anim. Sci. 86:3146–3158. doi:10.2527/jas.2008-0883. 18567723

[CIT0015] Sun, P., J. Q. Wang, and H. T. Zhang. 2010. Effects of *Bacillus subtilis* natto on performance and immune function of preweaning calves. J. Dairy Sci. 93:5851–5855. doi:10.3168/jds.2010-3263. 21094758

[CIT0016] Swanson, K. C., J. J. Gaspers, F. A. Keomanivong, T. C. Gilbery, G. P. Lardy, M. L. Bauer, and G. L. Stokka. 2018. Influence of feeding direct-fed microbial supplementation on growth performance and feeding behavior in naturally fed and conventionally fed finishing cattle with different dietary adaption periods. J. Anim. Sci. 96:3370–3380. doi:10.1093/jas/sky194. 29788297 PMC6095255

[CIT0017] Teo, A. Y. L., and H. M. Tan. 2005. Inhibition of Clostridium perfringens by a novel strain of *Bacillus subtilis* isolated from the gastrointestinal tracts of healthy chickens. Appl. Environ. Microbiol. 71:4185 –44190. doi:10.1128/AEM.71.8.4185-4190.2005. 16085801 PMC1183296

[CIT0018] Teo, A. Y. L., and H. M. Tan. 2006. Effect of *Bacillus subtilis in* PB6 (CLOSTAT) on broilers infected with a pathogenic strain of *Escherichia coli*. J. Appl. Poult. Res. 15:229–235. doi:10.1093/japr/15.2.229.

[CIT0019] Vasconcelos, J. T., N. A. Elam, M. M. Brashears, and M. L. Galyean. 2008. Effects of increasing dose of live cultures of *Lactobacillus acidophilus* (Strain NP 51) combined with a single dose of *Propionibacterium freudenreichii* (Strain NP 24) on performance and carcass characteristics of finishing beef steers. J. Anim. Sci. 86:756–762. doi:10.2527/jas.2007-0526. 18042817

[CIT0020] Wilson, B. K., B. P. Holland, D. L. Step, M. E. Jacob, D. L. VanOverbeke, C. J. Richards, T. G. Nagaraja, and C. R. Krehbiel. 2016. Feeding wet distillers grains plus solubles with and without a direct-fed microbial to determine performance, carcass characteristics, and fecal shedding of *Escherichia coli* O157:H7 in feedlot heifers. J. Anim. Sci. 94:2972/97–2972305. doi:10.2527/jas.2015-9601. 26812336

[CIT0021] Wilson, B. K., and C. R. Krehbiel. 2012. Current and future status of practical applications: beef cattle. In: T. R. Callaway and S. C. Ricke, editors. Direct-fed microbials and prebiotics for animals. New York: Springer Science & Business Media, p. 137–153. doi:10.1007/978-1-4614-1311-0.

[CIT0022] Wilson, B. K., C. J. Richards, D. L. Step, and C. R. Krehbiel. 2017. Best management practices for newly weaned calves for improved health and well-being. J. Anim. Sci. 95:2170–2182. doi:10.2527/jas.2016.1006. 28727007

[CIT0023] Wilson, B. K., D. L. Step, C. L. Maxwell, J. J. Wagner, C. J. Richards, and C. R. Krehbiel. 2015. Evaluation of multiple ancillary therapies used in combination with an antimicrobial in newly received high-risk calves treated for bovine respiratory disease. J. Anim. Sci. 93:3661–3674. doi:10.2527/jas.2015-9023. 26440032 PMC7199660

[CIT0024] Word, A., P. R. Broadway, N. Burdick-Sanchez, J. Carroll, K. Hales, K. Karr, B. Holland, G. Ellis, C. Maxwell, J. T. Leonhard, et al. 2022. The effect of supplementing CLOSTAT 500 (*Bacillus subtilis* PB6) to yearling steers in a commercial feedyard on health, *Salmonella* spp. prevalence, feedlot growth performance and carcass characteristics. Transl. Anim. Sci. 6:1–11. doi:10.1093/tas/txac131. PMC9661306 36381948

